# History of Alcohol Use Disorder and Housing Instability as Predictors of Fatigue and Mental Health Problems During the COVID-19 Pandemic

**DOI:** 10.1007/s11121-025-01784-0

**Published:** 2025-02-11

**Authors:** Noa Leiter, Jeremy W. Luk, Bethany L. Stangl, Tommy Gunawan, Melanie L. Schwandt, David Goldman, Nancy Diazgranados, Vijay A. Ramchandani

**Affiliations:** 1 Human Psychopharmacology Laboratory, National Institute on Alcohol Abuse and Alcoholism, Building 10 – CRC, Room 2-2352, Bethesda, MD 20892 USA; 2 Office of the Clinical Director, National Institute on Alcohol Abuse and Alcoholism, Building 10 – CRC, Room 1-5340, Bethesda, MD 20892 USA; 3 Laboratory of Neurogenetics, National Institute on Alcohol Abuse and Alcoholism, Rockville, MD USA

**Keywords:** Alcohol, Stress, Coping, COVID-19, Fatigue, Housing instability, Mental health, Psychopathology

## Abstract

Mental health and alcohol problems are significant public health concerns amid the COVID-19 pandemic. Housing instability and symptoms of fatigue are understudied aspects of the pandemic. This study examined history of Alcohol Use Disorder (AUD), history of COVID-19 infection, and housing instability as correlates of fatigue, anxiety, and depression symptoms. Data were drawn from 250 adults enrolled in an online survey within the NIAAA COVID-19 Pandemic Impact on Alcohol Study in between April 6 and June 2 of 2022. Participants completed self-report measures of housing stability, fatigue, and mental health symptoms. Multivariable analyses controlling for age, sex, race, ethnicity, and household income were conducted. Individuals with a history of AUD reported higher mental fatigue, anxiety symptoms, and depressive symptoms when compared to those with no history of AUD. Individuals with “other” housing arrangements (not renting or owning) reported higher mental fatigue, pandemic fatigue, anxiety symptoms, and depressive symptoms relative to homeowners. Individuals who worried about not having a place to live in the past 6 months reported higher physical fatigue, mental fatigue, anxiety symptoms, and depressive symptoms when compared to individuals without housing worry. History of COVID-19 infection was neither associated with mental health nor fatigue symptoms. Housing instability, as captured by housing worry and having “other” housing arrangements, was associated with greater fatigue and mental health problems, even after controlling for household income. Housing instability uniquely contributed to mental health symptoms, warranting further research and targeted prevention and intervention efforts.

## Introduction

The COVID-19 pandemic negatively impacted health around the world in a myriad of ways. Specifically, mental health problems rose as a public health concern, with the global prevalence of anxiety and depression increasing by 25% during the first year of the COVID-19 pandemic (World Health Organization, 2022). Additionally, physical, mental, and pandemic fatigue increased as a threat to individuals’ short and long-term wellbeing (Krakowczyk et al., 2022; Rudroff et al., 2020; Townsend et al., 2020). Various factors, including history of AUD, history of COVID-19 infection, and housing instability, may increase risk for mental health problems and fatigue. Therefore, the goal of the present study was to examine these variables (history of AUD, history of COVID-19 infection, and housing instability) as correlates of symptoms of fatigue, anxiety, and depression. Given the public health relevance of symptoms of mental health problems and symptoms of fatigue in the context of the pandemic, it is critical to identify modifiable factors that may help guide prevention efforts.

Fatigue emerged as a prevalent issue during the COVID-19 pandemic (Azzolino & Cesari, 2022; Taylor et al., 2022). One proposed definition of fatigue in the context of COVID-19 is “*the decrease in physical and/or mental performance that results from changes in central, psychological, and/or peripheral factors*” (Rudroff et al., 2020)*.* Expanding upon the common distinction between mental and physical fatigue, pandemic fatigue became a household term during the pandemic. Pandemic fatigue can be conceptualized as feeling tired of dealing with the COVID-19 pandemic and associated protective behaviors, and has rarely been investigated alongside mental and physical fatigue (World Health Organization, 2020). Pandemic fatigue created significant challenges in managing COVID-19 and is likely to be a relevant obstacle in addressing future pandemics or other public health crises (Taylor et al., 2022).

COVID-19 infection may influence risk for fatigue and mental health symptoms. As of January 18, 2023, the Centers for Disease Control and Prevention’s (CDC) COVID Data Tracker documented over 100,000,000 cases of COVID-19 infection in the United States (Centers for Disease Control and Prevention, n.d.). In clinical studies of COVID-19 patients, some individuals report suffering from persistent fatigue symptoms. Research indicates that persistent fatigue is not uncommon, with 13% to 33% of individuals reporting fatigue 16–20 weeks post-symptom onset (Sandler et al., 2021), and that fatigue is independent of the severity of the initial infection (Townsend et al., 2020). Along with fatigue, individuals with a history of COVID-19 infection have been shown to be at increased risk for anxiety and depressive symptoms (Colizzi et al., 2022; Xie et al., 2022).

Alcohol use disorder (AUD) is another risk factor for numerous health problems (Rehm, 2011). The association between AUD and anxiety and depressive symptoms is well-studied, with AUD often co-occurring with each mental health disorder (Anker & Kushner, 2019; McHugh & Weiss, 2019). Negative emotionality is a key factor contributing to problematic drinking within the cycle of addiction (Koob & Volkow, 2016). Potential mechanisms through which AUD is linked to increased mental health problems include neurochemical changes (Yang et al., 2022), sleep disruption (Koob & Colrain, 2020), maladaptive coping (Turner et al., 2018), and social isolation (Luk et al., 2024). In contrast, very little published research that we are aware of has examined the association between AUD and mental and physical fatigue (McCallum et al., 2019), and none to our knowledge has examined this relationship in the context of the COVID-19 Pandemic. Individuals with AUD tend to experience higher rates of sleep disruption (He et al., 2019), stress (Sinha, 2022), and social factors such as loneliness (Luk et al., 2024), which are risk factors for fatigue (Jaremka et al., 2014; Kop & Kupper, 2016; Lichstein et al., 1997). Especially considering the changes in alcohol policies, increased liquor sales, and increased problematic drinking behaviors for some demographic groups during the COVID-19 pandemic (Kerr et al., 2022), an improved understanding of the association between problematic alcohol use and fatigue symptoms is needed.

Alongside health challenges, the COVID-19 pandemic heavily impacted the economy, leading to historic inflation, work disruptions, and increased financial stress (Cavallo, 2022; Gray et al., 2023; Luk et al., 2022a, 2022b; Pew Research Center, 2021). Individuals with low financial resources may face challenges meeting their basic needs, with one understudied area being housing instability. Housing instability exists on a continuum and describes a lack of security in a person’s shelter, including difficulty paying rent and frequent moving, that is often a result of high housing costs relative to income (Frederick et al., 2014; Office of Disease Prevention and Health Promotion, n.d.). The Consumer Financial Protection Bureau (2021) reported that in 2020 there were 8 million renters behind on rent and a 250% increase in households that had fallen behind at least three months on mortgage payments. Recent census data similarly revealed increased housing instability in the United States during the COVID-19 pandemic (United States Census Bureau, 2022).

As housing instability was exacerbated during the pandemic, it would be valuable to examine the degree to which it conferred risk for mental health problems and fatigue symptoms. Generally, research indicates positive associations between housing instability and mental and physical health issues (Office of Disease Prevention and Health Promotion, n.d.; Rollings et al., 2022). In a cross-sectional study on hospitalizations using a national inpatient sample, Rollings and colleagues (2022) found that housing instability was associated with higher rates of admissions for behavioral, mental, and neurodevelopmental disorders, along with longer hospital stays and higher care costs. Additional research has shown a bi-directional relationship between homelessness and mental health issues (Padgett, 2020). Yet, very little equivalent research has investigated the associations between housing instability and fatigue symptoms, with one study finding higher rates of fatigue in individuals experiencing homelessness when compared to the general population (Gonzalez & Tyminski, 2020). Housing instability may increase risk for fatigue symptoms through similar hypothesized mechanisms as AUD, including sleep disruption (Bozick et al., 2021; Gonzalez & Tyminski, 2020) and stress (Kocalevent et al., 2011).

### The Current Study

The present study aimed to address these gaps in the literature by examining history of AUD, history of COVID-19 infection, and housing instability as risk factors for symptoms of fatigue, anxiety, and depression. First, we investigated the associations between each risk factor and outcome variable separately. Then, we examined the combined effects of these risk factors on mental health and fatigue outcomes, controlling for the covariates of age, sex, race, ethnicity, and household income. We hypothesized that after adjusting for covariates, history of AUD, history of COVID-19 infection, and housing instability would be associated with higher levels of mental, physical, and pandemic fatigue. We further hypothesized that similar patterns would be observed for anxiety and depressive symptoms. Understanding potential risk factors for the development of fatigue and mental health may guide targeted prevention and policy efforts that better address the needs of those at higher risk.

## Methods

### Participants

Data were drawn from the National Institute on Alcohol Abuse and Alcoholism COVID-19 Pandemic Impact on Alcohol Study (C19-PIA Study), a longitudinal study that follows participants over a 2-year period. The baseline characteristics of the larger cohort have been reported elsewhere (Luk et al., 2022, 2023). Of the enrolled participants, 265 opted to participate in an additional follow-up survey around the 2-year anniversary of the pandemic in between April 6 and June 2 of 2022. Fifteen participants were excluded due to missing data on either history of AUD (*n* = 4) or outcome variables of interest (*n* = 11), yielding an analytic sample of 250 participants. All study participants provided informed consent. The C19-PIA Study protocol was approved by the National Institutes of Health Intramural Institutional Review Board and is registered in clinicaltrials.gov (NCT04391816).

### Measures

*History of AUD and COVID-19 Infection.* AUD status was assessed using the Structural Clinical Interview for Diagnostic and Statistical Manual of Mental Disorders (First, 2015) and was obtained from the NIAAA natural history protocol database (Luk et al., 2022, 2023). History of COVID-19 infection was assessed using two questions: “Throughout the pandemic, how many times did you get tested for COVID-19?” and “Did you test positive for COVID-19?” Participants who were never tested for COVID-19 (*n* = 27, 10.8%) or did not test positive (*n* = 156, 62.4%) were coded as negative, and those who tested positive for COVID-19 were coded as positive (*n* = 67, 26.8%).

*Housing Instability.* Housing instability was evaluated with three questions. The first question assessed housing arrangement: “Do you own or rent the place where you live?” Response options included *own, rent,* and *other arrangements* (National Health Care for the Homeless Council (NHCHC), n.d.). The second question assessed housing length: “How many years have you lived at your current address?” Response options included *less than 1 year, 1 to 2 years, 3 to 5 years, 6 to 10 years, 11 to 20 years,* and *more than 20 years* (UK Biobank, n.d.). Responses were recoded into three categories: *less than two years, three to ten years,* and *eleven or more years*. The third question assessed housing worry: “In the past 6 months, have you been worried or concerned about NOT having a place to live?” Response options included *yes* and *no* (Montgomery et al., 2013).

*Fatigue.* Fatigue was evaluated using an adapted 10-item version of the Fatigue Assessment Scale (FAS; Michielsen et al., 2003). Physical fatigue was measured using three items from the original scale: “I am bothered by fatigue”, “I get tired very quickly”, and “Physically, I feel exhausted” (Cronbach’s α = 0.90). Mental fatigue was measured using three items from the original scale: “I have problems starting things”, “I have no desire to do anything”, “Mentally, I feel exhausted” (Cronbach’s α = 0.86). Pandemic fatigue was measured using four items created by the study team: “I am tired of social distancing”, “I am tired of mask wearing”, “I am tired of hearing and talking about COVID”, “COVID has increased my levels of fatigue” (Cronbach’s α = 0.82). Response options ranged from 1 (“Never”) to 3 (“Regularly”) and 5 (“Always”). Mean scores were computed for each of these subscales for analyses.

*Anxiety Symptoms.* The Generalized Anxiety Disorder Assessment (GAD-7; Spitzer et al., 2006) is a seven-item measure assessing anxiety symptoms over the previous two weeks. Each item response ranges from 0 (“not at all”) to 3 (“nearly every day”), yielding a maximum sum score of 21. The Cronbach’s α of the GAD-7 in the current sample was 0.92.

*Depressive Symptoms.* The Patient Health Questionnaire (PHQ-9; Kroenke et al., 2001) is a nine-item measure assessing depressive symptoms over the previous two weeks. Each item response ranges from 0 (“not at all”) to 3 (“nearly every day”), yielding a maximum sum score of 27. The Cronbach’s α of the PHQ-9 in the current sample was 0.90.

*Demographic Characteristics.* Participants reported their age, sex, race, and ethnicity, which were included as covariates. Household income was also self-reported and included as a covariate to ensure that the effects of housing stability on outcomes were independent of household income more generally.

### Statistical Analysis

Univariable regression analysis was used to examine the association between risk factors (history of AUD, history of COVID-19 infection, housing instability) and outcomes (fatigue, anxiety, depression) separately. Then, multivariable regression models adjusting for age, sex, race, ethnicity, and household income were used to examine the association of history of AUD, history of COVID-19 infection, and housing instability with symptoms of fatigue, anxiety, and depression. All analyses were conducted in STATA 18 (StataCorp, 2023 College Station, TX).

## Results

Table 1 shows the demographic characteristics of the study sample. Participants had a mean age of 47.3 years (SD = 14.6) and were 50% female (*n* = 125) and 50% male (*n* = 125). The study sample was racially diverse, with 52.8% White (*n* = 132), 31.6% Black/African American (*n* = 79), and 15.6% Other Race (*n* = 15.6). In terms of ethnicity, 8.0% (*n* = 20) self-identified as Hispanic or Latino. Ranges of annual household income were less than $19,999 for 23.6% of participants (*n* = 59), in between $20,000 and $74,999 for 30.4% of participants (*n* = 76), in between $75,000 and $149,999 for 26.8% of participants (*n* = 67) and $150,000 or more for 19.2% of participants (*n* = 48). Of the 250 participants, 26.8% (*n* = 67) reported testing positive for COVID-19 infection and 32.0% (*n* = 80) had a positive history of AUD.

**Table 1:** Demographic characteristics of the overall analytic sample

	n	%
Sex		
Female	125	50.0%
Male	125	50.0%
Race		
White	132	52.8%
Black	79	31.6%
Other	39	15.6%
Ethnicity		
Non-Hispanic	221	88.4%
Hispanic	20	8.0%
Unknown	9	3.6%
Income		
$19,999 or less	59	23.6%
$20,000—$74,999	76	30.4%
$75,000—$149,999	67	26.8%
$150,000 or more	48	19.2%
Housing Arrangement		
Own	97	38.8%
Rent	130	52.0%
Other Arrangements	23	9.2%
Housing Length		
Less than two years	103	41.2%
Three to ten years	80	32.0%
Eleven or more years	67	26.8%
Housing Worry		
No	206	82.4%
Yes	44	17.6%
Positive COVID-19 Infection	67	26.8%
History of AUD	80	32.0%

**Fig. 1:**
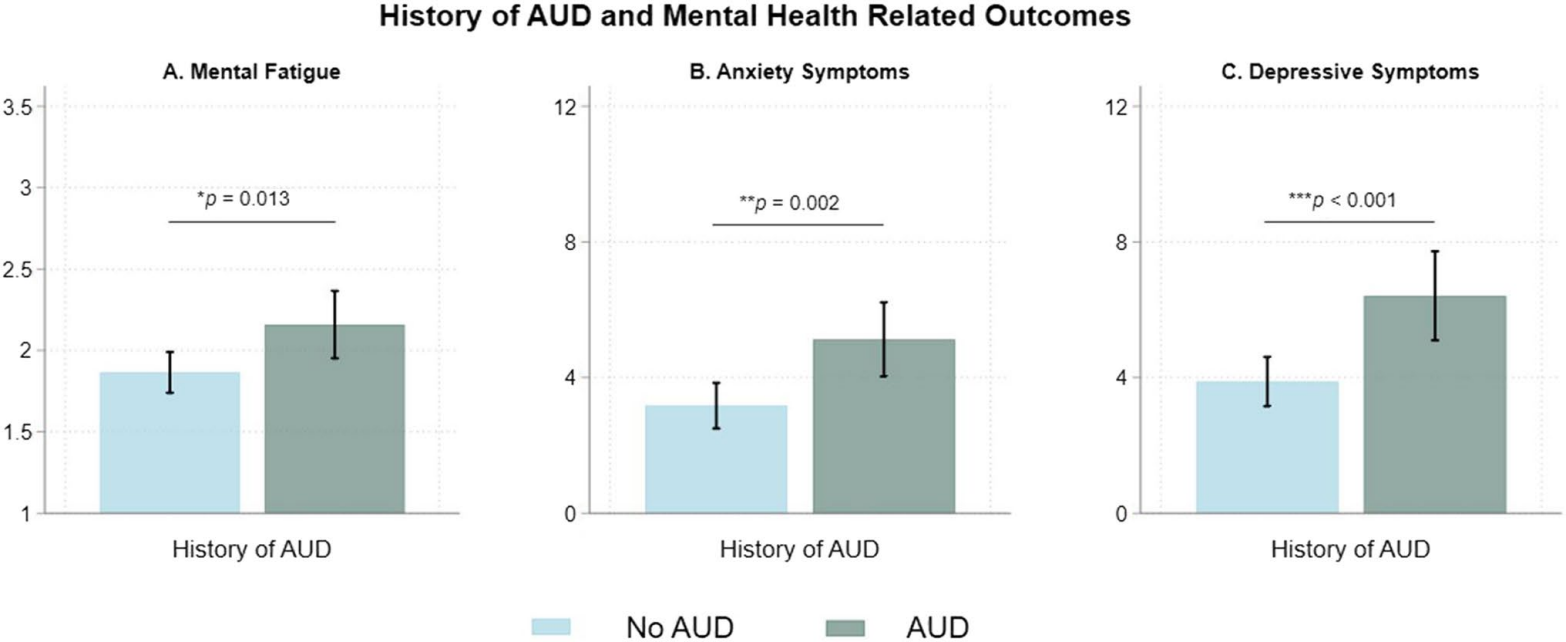
History of AUD and mental health related outcomes. The bar graphs show mean mental fatigue, and the total scores for anxiety and depressive symptoms for individuals with a history AUD and individuals without a history of AUD. Error bars represent 95% confidence intervals

**Fig. 2:**
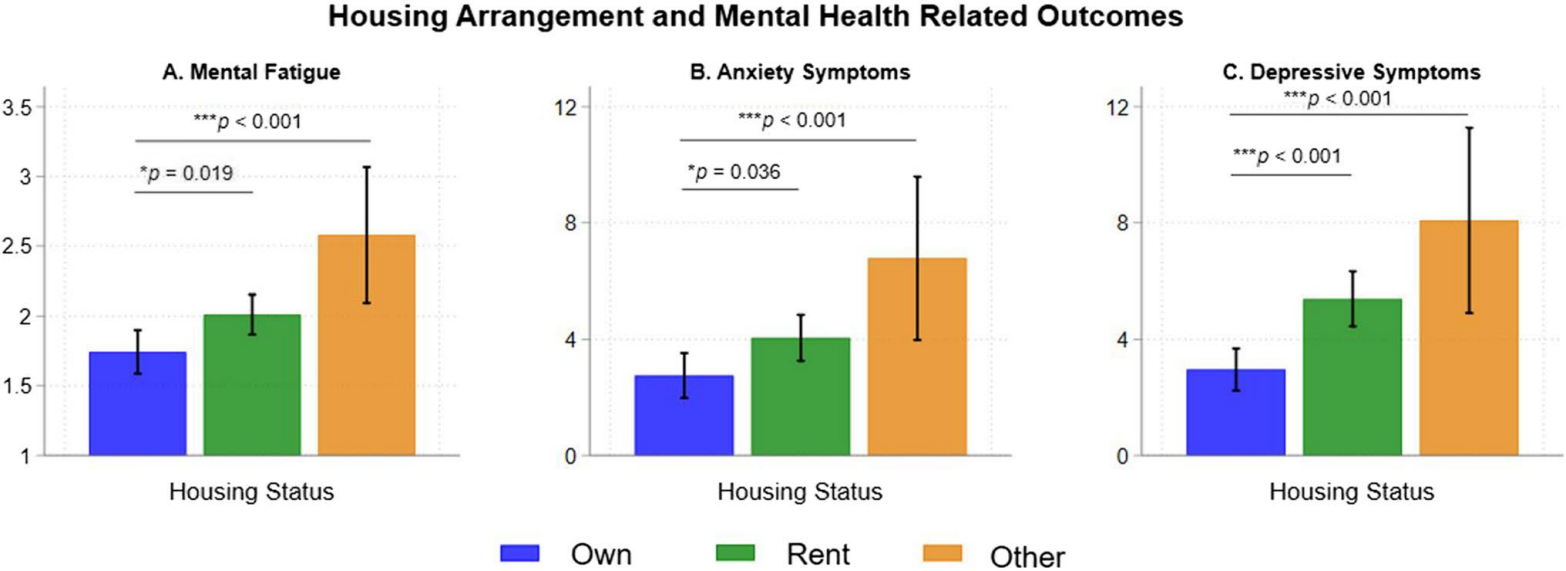
Housing arrangements and mental health related outcomes. The bar graphs show mean mental fatigue, and the total scores for anxiety and depressive symptoms for individuals reporting owning a house, renting a house, and experiencing other living arrangements. Error bars represent 95% confidence intervals

**Fig. 3:**
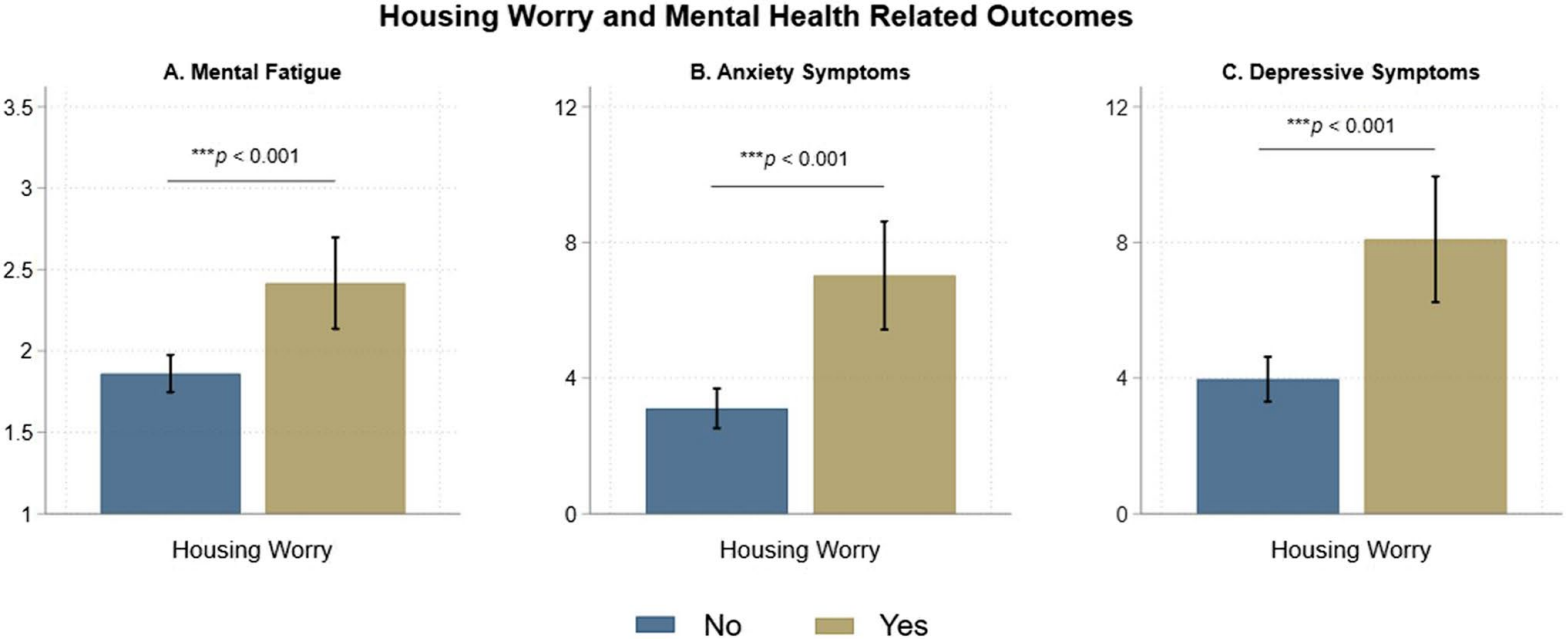
Housing worry and mental health related outcomes. The bar graphs show mean mental fatigue, and the total scores for anxiety and depressive symptoms for individuals experiencing housing worry and those not experiencing housing worry. Error bars represent 95% confidence intervals

Figure 1 shows that individuals with a history of AUD reported higher mental fatigue (Mean = 2.16 versus 1.86, *p* = 0.013), anxiety symptoms (Mean = 5.12 versus 3.17, *p* = 0.002), and depressive symptoms (Mean = 6.41 versus 3.88, *p* < 0.001) than those without a history of AUD. Figure 2 shows that renters reported higher mental fatigue (Mean = 2.01 versus 1.74, *p* = 0.019), anxiety symptoms (Mean = 4.05 versus 2.75, *p* = 0.036), and depressive symptoms (Mean = 5.38 versus 2.96, *p* < 0.001) when compared to individuals who reported owning a house. Individuals experiencing “other” housing arrangements also reported higher mental fatigue (Mean = 2.58 versus 1.74, *p* < 0.001), anxiety symptoms (Mean = 6.78 versus 2.75, *p* < 0.001), and depressive symptoms (Mean = 8.09 versus 2.96, *p* < 0.001) when compared to individuals who reported owning a house. Housing length was not associated with the clinical outcomes. Figure 3 shows that individuals with housing worry reported higher mental fatigue (Mean = 2.42 versus 1.86, *p* < 0.001), anxiety symptoms (Mean = 7.08 versus 3.11, *p* < 0.001), and depressive symptoms (Mean = 8.09 versus 3.97 *p* < 0.001) than individuals without housing worry.

**Fig. 4:**
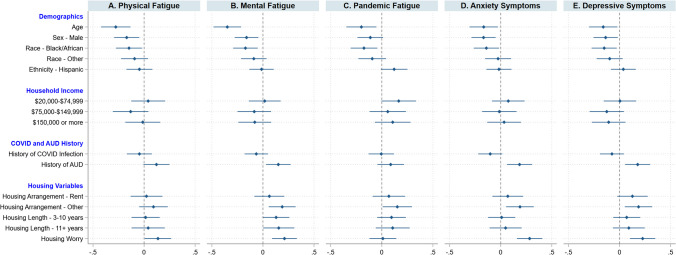
Coefficients plot illustrating results from the multivariable linear models. Effects of demographic, household income, history of COVID-19 infection, history of AUD, and housing variables on physical fatigue, mental fatigue, pandemic fatigue, anxiety symptoms, and depressive symptoms, via multivariable regression analyses. Standardized regression coefficients, and 95% confidence intervals, are presented. The referent groups for sex, race, and ethnicity were female, White, and not Hispanic/Latino, respectively

In multivariable analyses, the effects of renting on clinical outcomes were attenuated, whereas all other associations persisted after adjusting for age, sex, race, ethnicity, and household income. Figure 4 shows regression coefficient plots illustrating the estimated main effects of COVID-19 infection, history of AUD, housing instability indicators on three types of fatigue (physical, mental, and pandemic), anxiety symptoms, and depressive symptoms while adjusting for covariates. Individuals with a history of AUD reported higher mental fatigue (*b* = 0.27, 95% CI = 0.05, 0.50), anxiety symptoms (*b* = 1.85, 95% CI = 0.60, 3.09) and depressive symptoms (*b* = 2.05, 95% CI = 0.64, 3.45) when compared to those with no history of AUD. Individuals with “other” housing arrangements (not renting or owning) reported higher mental fatigue (*b* = 0.57, 95% CI = 0.17, 0.96), pandemic fatigue (*b* = 0.53, 95% CI = 0.03, 1.04), anxiety symptoms (*b* = 3.06, 95% CI = 0.87, 5.26) and depressive symptoms (*b* = 3.42, 95% CI = 0.95, 5.89) relative to homeowners. Individuals with housing worry reported higher physical fatigue (*b* = 0.32, 95% CI = 0.14, 0.62), mental fatigue (*b* = 0.49, 95% CI = 0.21, 0.77), anxiety symptoms (*b* = 3.49, 95% CI = 1.95, 5.03), and depressive symptoms (*b* = 3.13, 95% CI = 1.40, 4.87) when compared to individuals without housing worry. No significant differences in clinical outcomes were found between individuals reporting a positive history of COVID-19 infection and no history of COVID-19 infection.

## Discussion

The present study examined the associations between three potential risk factors (history of AUD, history of COVID-19 infection, and housing instability) and symptoms of fatigue, anxiety, and depression. Alcohol Use Disorder (AUD) commonly co-occurs, either simultaneously or sequentially, with other mental health disorders (Kwako et al., 2019; Shivani et al., 2002). In the present study, both univariable and multivariable analyses revealed that history of AUD was associated with outcomes related to mental health, including increased mental fatigue, anxiety symptoms, and depressive symptoms. History of AUD was not associated with physical or pandemic fatigue. One explanation for the association between alcohol use problems and poor mental health is maladaptive coping. Theoretical frameworks such as the self-medication hypothesis and the tension reduction theory posit that individuals may use alcohol as a way to cope and attain relief from stress or negative emotions (Greeley & Oei, 1999; Khantzian, 1985; Lac & Luk, 2023; Turner et al., 2018). This may be of particular relevance in the context of the COVID-19 pandemic, as individuals who increased problem drinking during the pandemic were more likely to report drinking to cope with pandemic related issues, such as increasing levels of social disconnect (Mohseni et al., 2022).

Prior research indicates that COVID-19 infection may result in symptoms of fatigue, lasting weeks or months after infection (Sandler et al., 2021; Townsend et al., 2020), as well as increase risk for negative mental health symptoms (Colizzi et al., 2022; Xie et al., 2022). Contrary to these findings, results in the present study indicate that history of COVID-19 infection was not associated with measures of fatigue or mental health. This may reflect the shifting nature of the pandemic over time, including the varying long-term effects of COVID-19 depending on severity of infection, timing, and virus strain. Specifically, participants in the C19-PIA Study may have experienced milder cases of COVID-19, as most reported COVID-19 cases occurred after January 2022 (Luk et al., 2022, 2023). Lower rates of long COVID-19 linked with the Omicron variant, compared to previous variants such as Delta (Antonelli et al., 2022), along with increased availability of treatments like Paxlovid later in the pandemic, could have also accounted for these results. Additionally, our sample differed from much previous research that focused on hospitalized patients (Sandler et al., 2021). Despite nonsignificant findings, understanding the association of viral infections, like COVID-19, with physical and mental health is necessary to inform post-infection care. Social determinants of health, such as housing instability, should be considered in the development of care strategies, especially considering that housing instability is a risk factor for COVID-19 infection. For instance, studies have shown that individuals experiencing housing insecurity or homelessness have been found two-times more likely to experience COVID-19 re-infection when compared to individuals with stable housing (Bean et al., 2021; Rozenfeld et al., 2020).

Along with increased risk for COVID-19 infection, housing instability is associated with a myriad of other physical and mental health challenges (Burgard et al., 2012; Padgett, 2020; Rollings et al., 2022). In the present study, housing instability was generally positively associated with fatigue and mental health symptoms. Reporting “other” housing arrangements was associated with increased mental fatigue, anxiety symptoms, and depressive symptoms. Similarly, housing worry was associated with higher mental and physical fatigue, along with anxiety and depressive symptoms. These findings corroborate literature linking housing instability and housing disruptions with negative mental health (Gray et al., 2023; Padgett, 2020; Pevalin, 2009; Rollings et al., 2022) and extend prior research by looking at the associations between housing instability indicators and symptoms of fatigue. By investigating housing status in conjunction with housing worry we have captured both individuals’ objective housing arrangements and their perception of the stability of these arrangements (housing worry). Our results illustrate that housing instability uniquely contributes to negative mental and physical health and points to the need for effective prevention and intervention strategies.

Housing instability is of relevance to prevention science and is critical to examine in the context of AUD given the link between experiencing homelessness and alcohol use disorder in the United States (Collins et al., 2021). Alcohol use problems have been established as contributors to the initiation and maintenance of homelessness (McVicar et al., 2015; North et al., 1998). Epidemiological studies have revealed that approximately 60% of individuals experiencing homelessness have a lifetime history of AUD (Asana et al., 2018; North et al., 2010), which is notably higher than the general US population (Grant et al., 2015; McVicar et al., 2015; North et al., 1998). The dissemination of accessible and effective treatment of AUD, such as behavioral and pharmacological harm-reduction interventions, may work to reduce rates of AUD and alcohol related mortality in this population (Collins et al., 2021). Particular attention may be needed for individuals with AUD and comorbid mood and anxiety disorders, as this population is more likely to report financial barriers to treatment services (Kaufmann et al., 2014).

To mitigate the severe impact of the COVID-19 pandemic on housing stability, the United States government instituted nearly $47 billion in emergency rental assistance and increased tenant protections (Vasquez et al., 2022). However, as these funds run out and the increased protections expire, it is important to consider how initiatives may be continued to support housing stabilization to prevent mental health symptoms. One evidence-based model of intervention is *Housing First*, which uses a recovery-oriented approach that prioritizes connecting individuals and families to quick and stable housing, without any prerequisites such as sobriety or program completion (National Alliance to End Homelessness, 2022). This model has been shown to be successful in populations of individuals experiencing alcohol use problems (Asana et al., 2018; Collins et al., 2013). Macro-level factors, such as housing stability, should be integrated into personalized intervention efforts to target quality of life concerns and facilitate long-term recovery (Luk & Thompson, 2024; Witkiewitz & Tucker, 2024). Additionally, services and interventions targeting mental health related outcomes can also be incorporated into housing stability efforts given that integrated approaches have been shown to lead to more favorable outcomes (McHugo et al., 2004). Our findings should be replicated in population-based studies to better inform policy implications, including ways to support and increase access to affordable housing and community-based rehabilitation services.

From a prevention science perspective, more nuanced measurement of housing instability is critical to understand what aspect of housing problems conferred the most risk for mental health outcomes. In existing literature, the conceptualization of housing instability tends to be narrow. Previous studies investigating the associations between housing instability and health outcomes have focused on specific types of housing instability, such as housing payment problems (Taylor et al., 2007), eviction (Pevalin, 2009), foreclosure (Pollack & Lynch, 2009), and homelessness (Funk et al., 2022; Liu & Hwang, 2021). Other researchers have studied housing instability by focusing on certain groups within the population, such as renters (Kim & Burgard, 2022), mothers (Suglia et al., 2011) or cancer survivors (Coughlin & Datta, 2022). Given this background, a key strength of the present study was our ability to assess for multiple aspects of housing instability, including individuals’ housing arrangements (rent, own, other), housing length, and housing worry. Both housing arrangement and housing length tap into the objective security of an individuals’ housing, as they reflect the stability and permanence of their living situation. Housing worry captures the subjective experience of concern or anxiety regarding housing status. Put together, these three questions provide a comprehensive assessment of the housing instability construct.

Despite this strength and the timeliness of the research topic, this study has several limitations. First, while we statistically controlled for household income in the multivariable analyses, which ensured that housing instability was not confounded by socioeconomic resources, other potential confounding variables related to daily living, such as employment, food insecurity, and challenges with transportation, were not included. Second, the psychometric properties of the pandemic fatigue items have not been validated previously. Hence, the relatively weaker findings on pandemic fatigue are difficult to interpret as they may reflect potential measurement bias. Third, the data analyzed were cross-sectional, limiting our ability to draw inferences about the direction of effects. Therefore, it is possible that symptoms of fatigue or mental health problems could have increased risk for AUD (e.g., through coping) or housing instability (e.g., due to unemployment), rather than the other way around (Castro-Marrero et al., 2019; Olesen et al., 2013; Turner et al., 2018). Fourth, the use of a convenience sample and the small sample size limited generalizability of findings. Fifth, the data were based on self-report, which can introduce recall and social desirability biases. To address these limitations, longitudinal research can be utilized to clarify temporal ordering and potential bi-directional effects of fatigue, housing instability, and mental health symptoms across time, as well as elucidate potential mechanisms that may explain these associations.

In conclusion, this study revealed that housing instability, as captured by housing worry and having “other” housing arrangements, was associated with greater mental and physical fatigue, as well as mental health problems. History of AUD was also shown to increase risk for mental fatigue and anxiety and depressive symptoms. These findings have implications for prevention science such as highlighting the importance of intervening at the environmental level and targeted services offered to those with a history of AUD. Considering the negative impact of economic uncertainties and inflation on people’s livelihood and the exacerbated housing conditions during the COVID-19 pandemic, there is an urgent need to research on the detrimental health implications of housing instability and the potential benefit of implementing interventions aimed at increasing housing stability in vulnerable groups.
